# Author's reply:

**DOI:** 10.1192/bjb.2021.96

**Published:** 2021-12

**Authors:** Gethin Morgan

**Affiliations:** Emeritus Professor of Mental Health, University of Bristol, Bristol, UK. Email: hilary.howard@blueyonder.co.uk

17 June

I am grateful to Drs Albert, Gallen and Gaur for their interest in my paper. Unfortunately they appear to have misunderstood some major points which I make. I certainly do not suggest that the assessment of suicidal thoughts is futile in short-term prediction of suicide. I argue exactly the opposite, presenting evidence that provided this is carried out correctly and appropriately, it should have significant predictive value.

What is more, I do not in any way suggest that ongoing care of suicidal patients should be handed back to the general practitioner (GP), certainly not before their problems have been resolved. My suggested letters are meant as clinical summaries which should be sent routinely to GPs by any psychiatric team as part of good ongoing clinical care. They do not mean, in any way, that the secondary service thereby should relinquish ongoing clinical care of their patients before treatment is complete.

How to maintain good ongoing supportive care of patients who have experienced a suicidal crisis is an important clinical challenge. My paper considers how the psychiatrist might attempt to achieve this, by emphasising concern to provide the form of help which would be most acceptable to the patient, and to which he/she would readily turn should the crisis recur.

